# Natural Fibre Composites and Their Mechanical Behaviour

**DOI:** 10.3390/polym15051185

**Published:** 2023-02-26

**Authors:** Mariana Doina Banea

**Affiliations:** 1 Federal Center of Technological Education in Rio de Janeiro, Rio de Janeiro 20271-110, Brazil; doina.banea@cefet-rj.br or mbanea@ua.pt; 2 CICECO—Aveiro Institute of Materials, Department of Materials and Ceramic Engineering, University of Aveiro, 3810-193 Aveiro, Portugal

At present, natural-fibre-reinforced-composites (NFRCs) are seen as realistic alternatives to synthetic- (e.g., glass) fibre-reinforced composites in many applications. Natural fibres (e.g., sisal [1], ramie [1], hemp [2], curauá [1], bamboo [3], kenaf [4,5], flax [6], jute [7], etc.) have attracted the attention of researchers for their application in several industries, such as automotives, construction and furniture, sports, and music instruments, among others. The lower weight and relatively lower cost of natural fibres are the main reasons for their use in composites in these applications. Another relevant factor in using natural fibres as reinforcement materials in composites is their eco-friendliness associated with the lower energy consumption in their manufacturing process. However, their recyclable properties also depend on the type of matrices used in composite production.

The main objective of this Special Issue was to provide a platform for the dissemination of the latest scientific and technical advancements in the optimization of the mechanical properties, durability, processing, and applications of natural-fibre-reinforced composites.

Natural-fibre-reinforced composites vary greatly in their mechanical properties. Mechanical properties (e.g., tensile, flexural, and impact) are highly dependent on different factors such as fibre and matrix type, interfacial bonding between fibre and matrix, fibre dispersion and orientation, and processing, among others. By increasing their mechanical performance, the capabilities and applications of natural-fibre-reinforced composites will be expanded.

One method to increase the mechanical performance of NFRCs to broaden their applications is hybridization [1]. Generally, natural-fibre-reinforced hybrid composites are produced by hybridizing natural fibres with another either natural or synthetic fibres with superior properties (e.g., higher mechanical strength, chemical stability, nontoxicity, resistance to high temperatures, and thermal or acoustic insulation). Pereira et al. [1] investigated the influence of hybridisation on the mechanical and thermal properties of intralaminar natural-fibre-reinforced hybrid composites. They concluded that the mechanical properties are improved via the hybridization of sisal-based composites with ramie, sisal, and curauá fibres. On the other hand, they stated that hybridization did not significantly affect the thermal stability of the composites studied.

Alonso-Montemayor et al. [2] studied natural-fibre-reinforced composites using 10, 20, and 30% bleached hemp fibres (by weight) into the polyamide 6 polymer matrix structure. They stated that it is possible to obtain tensile strengths higher than glass-fibre-reinforced polyolefin and explained that this effect was due to the strong adhesion between the fibres and the polymer matrix and the good distribution of the fibres into the composite.

It is known that the thermal stability of natural-fibre-reinforced composites is a relevant aspect to be considered as the processing temperature plays a crucial role in the fabrication process. At higher temperatures, the natural fibre components (e.g., cellulose, hemicellulose, and lignin), start to degrade, and the major properties (mechanical and thermal) of the composite change. Neto et al. [8] presented an overview of the recent advancements made regarding the thermal properties of natural- and hybrid-fibre-reinforced composites in thermoset and thermoplastic polymeric matrices. The methods used to determine the thermal properties of natural and hybrid composites along with the main factors that affect the thermal properties of natural and hybrid fibre composites (fibre and matrix type, the presence of fillers, fibre content and orientation, the treatment of the fibres, and manufacturing process) were discussed. They stated that it is crucial to ensure that the natural fibres used in composites can withstand the heat required during the fabrication process and retain their characteristics in service.

da Silva et al. [5] studied the thermal properties and ballistic performance of kenaf-fibre-reinforced epoxy composites and concluded that the composites reinforced with 30 vol.% kenaf fibre presented the best results. Neves et al. [9] investigated the ballistic behaviour of uni- and bidirectional pineapple leaf fibre (PALF)-reinforced epoxy composites functionalized with graphene oxide (GO). They found that bidirectional GO non-functionalized PALF fibres in a GO-reinforced matrix showed the best results, indicating its possible application as a second layer in multilayered armour systems.

Ahmad-Saffian et al. [4] studied the possibility of improving the mechanical, thermal, and electrical properties of kenaf-fibre-reinforced composites via the incorporation of 30% lignin in polypropylene (PP) matrix composites. They found improved thermal stability and higher thermal diffusivity compared to pure PP, but the tensile strength decreased. It was concluded that further studies are necessary in order to improve the adhesion between the PP polymer matrix and kenaf core fibres and lignin.

The main natural fibres studied and used in the industry (e.g., jute, sisal, kenaf, and flax) are well-established on the global market with a well-defined production line. However, new promising types of natural fibres are being discovered and studied. For instance, Neuba et al. [10] investigated sedge fibres from the seven-islands-sedge plant (Cyperus malaccensis), while Souza et al. [11] studied the properties of Caranan fibre (Mauritiella armata) for possible application as a natural fibre reinforcement in composite materials. Nevertheless, some improvements are needed in their production line to be more commercially affordable and to enable their widespread use.

Both thermoplastic and thermoset polymers are used as matrices in natural-fibre-reinforced polymer composites. There is an increased interest in the scientific community regarding the use of bio-based polymers in composites, as combining these matrices with natural fibres produces “green composites” or “bio-composites”. Bolcu and Stănescu [6] used three types of hybrid matricies based on the Dammar natural hybrid resin and studied the mechanical and chemical properties of flax-fibre-reinforced composite materials, while Dolza et al. [7] used bio-based high-density polyethylene (BioHDPE) to fabricate green composites reinforced with short flax, hemp, and jute fibres. However, the price of these bio-based matrices is still higher than their corresponding petroleum-based counterparts.

Finally, another area currently undergoing rapid development is the application of natural fibres as reinforcements in composites produced via additive manufacturing (AM or 3D printing). This technology allows for the fabrication of complex geometries without the need for expensive tooling and moulds. The use of natural fibres as filament reinforcements was investigated by Cavalcanti et al. [12]. Short curauá fibres with different lengths (3, 6, and 8 mm) and concentrations in terms of weight percentage (2, 3.5, and 5 wt. %) were used to fabricate polylactic acid (PLA) filaments which were subsequently used to fabricate composites via fused deposition modelling. They concluded that curauá-fibre-reinforced PLA composites may be a promising innovation to improve the performance of these materials, which might enable them to be used in new applications. However, some challenges remain to be solved in the fabrication of the reinforced filaments, such as difficulty in quality control due to voids and porosities, among others.

To summarize, this subject is vast and cannot be collected in a single volume. However, this Special Issue provides readers with a broad and updated overview of this topic.

## Data Availability

Not applicable.
